# Determinants of long-lasting insecticidal net ownership and utilization in malaria transmission regions: evidence from Zimbabwe Demographic and Health Surveys

**DOI:** 10.1186/s12936-019-2912-x

**Published:** 2019-08-20

**Authors:** Oscar Tapera

**Affiliations:** Department of Mathematics, Faculty of Science, Midlands State University, Gweru, Zimbabwe

**Keywords:** Malaria, Long-lasting insecticidal nets, Indoor residual spraying, Utilization, Universal coverage, Zimbabwe

## Abstract

**Background:**

Long-lasting insecticidal nets (LLINs) were first introduced in 2010 as a vector control intervention, to complement indoor residual spraying, to reduce malaria transmission in Zimbabwe. The objective of this study was to investigate factors that were associated with LLIN ownership and utilization among households in malaria transmission regions of Zimbabwe.

**Methods:**

A secondary analysis of cross sectional data from the Zimbabwe demographic and health survey (ZDHS) conducted in 2010 and 2015 surveys round was conducted. The analysis used household-level datasets from across the country to generate evidence for the study. Univariate analysis was used to yield descriptive statistics. Principal component analysis (PCA) was used to calculate wealth quintiles. Binary logistic regression approach was used to identify determinants of LLIN ownership and utilization after controlling for other factors. Data analyses were conducted using *STATA* version 14 software.

**Results:**

There were no major changes in demographic characteristics of households sampled between 2010 and 2015 survey cycles. LLIN ownership increased significantly by 42 percentage points from 2010 to 2015. There was a tremendous increase in universal coverage of LLINs between 2010 and 2015. The overall utilization levels of LLINs among children under-5 years decreased by 11 percentage points between 2010 and 2015. LLIN usage amongst households followed the same trend with that of the under-fives. Using logistic regression model for 2015 data, region/province, type of place of residence, availability of electricity, radio, roof type, gender of head of household, having telephone, type of cooking fuel, presence of mobile phone, owning a bank account, IRS spraying in the previous 12 months, wealth index, and satellite television decorder were independently associated with net ownership among households. Type of place of residence, age of household head, type of cooking fuel, IRS in previous 12 months, and pregnancy were associated with LLIN utilization.

**Conclusion:**

This study revealed increasing LLIN coverage and low usage in malaria-transmission regions of Zimbabwe. Strengthening of LLIN campaigns, social behaviour change communication (SBCC) interventions and programme routine monitoring are recommended.

## Background

Malaria continues to be a significant public health threat in developing countries [1]. It is the third highest cause of morbidity and mortality in Zimbabwe and it accounts for 30% of total outpatient attendance and 12% of all hospital admissions [2]. More than 50% of the population in Zimbabwe lives in malaria-transmission areas. The transmission of the disease is unstable in the country with around 51 of the 63 districts considered malarious  [2]. Recent data have shown an increase in cases and outbreaks of malaria due to the incessant rains that the southern African region experienced [3]. Some of the most vulnerable population groups include pregnant women, children under 5 years old, and people living with HIV/AIDS (PLWHA). Most of the malaria cases are caused by *Plasmodium falciparum* and the major vector is *Anopheles arabiensis* [4].

Zimbabwe’s malaria control programme mainly uses vector control measures, such as indoor residual spraying (IRS) and distribution of long-lasting insecticide-treated nets (LLINs) to reduce malaria incidence in endemic zones. Case management, prompt access to diagnosis and treatment and intermittent preventive treatment in pregnancy (IPTp) are other strategies used to reduce malaria transmission [1, 2]. Zimbabwe has a history of using IRS since the 1940s, using dichloro-diphenol-trichloro-ethane (DDT) and more recently pyrethroids and organophosphates. Historically, IRS was used in malaria control until the first LLIN mass campaign in 2010 [5]. LLINs are one of the integrated vector control management tools recommended for prevention and control of malaria in Zimbabwe. Since 2010, the country distributed predominantly LLINs and the distributions were focused on districts with only low to moderate malaria transmission. The mass distribution strategy targets one net per sleeping space or sleeping pair [6]. Universal coverage of vector control strategies is required to achieve malaria incidences fewer than 1 per 1000, which are the levels considered for malaria elimination [1]. Coverage of IRS or LLINs of at least 80% by population at risk should be achieved and maintained to reduce malaria burden significantly [5].

LLINs have been routinely distributed through mass campaigns; however, according to the Zimbabwe demographic and health survey (ZDHS) [6], about 48% of households owned at least one LLIN in malaria-transmission zones in Zimbabwe. Nationally, 37% of the de facto population had access to an LLIN [6]. These statistics show huge ownership gaps, which need improvement to achieve significant vector control coverage. Recently, Ministry of Health and Child Care (MoHCC) and its partners piloted routine LLIN distributions in order to complement mass campaigns in improving LLIN coverage rates. The target was to ensure and maintain a 95% LLIN coverage rate in transmission zones by 2015  [7]. World Health Organization defined adequate LLIN coverage as one LLIN per sleeping space or sleeping pair [8]. With increased LLIN coverage rates it is anticipated that malaria burden will be reduced significantly. This study was envisaged to understand some of the factors associated with LLIN ownership and usage in malaria transmission regions of Zimbabwe.

## Methods

Data from ZDHS 2010 and 2015 cycles were requested online from ICF International and permission to use the datasets was obtained after outlining the purpose of the request. Demographic and health surveys are implemented by ICF International and are funded by the USAID. Over the years ICF International has provide technical assistance to more than 300 surveys in at least 90 countries across the globe. The Demographic and Health Survey Programme has earned a worldwide reputation for collecting and disseminating accurate, nationally representative data on fertility, family planning, maternal and child health, gender, HIV/AIDS, malaria, and nutrition [9].

In the 2010–2011 ZDHS survey, a nationally representative sample of 9171 women aged 15–49 years and 7480 men aged 15–54 years in all selected households were interviewed. This represented a response rate of 93% for women and 86% for men. This sample provided estimates at the national, urban–rural and provincial levels [10]. A nationally representative sample of over 11,000 household members comprising women aged 15–49 and men aged 15–54 were eligible for individual interviews. The 2015, ZDHS is a follow-up survey to the 1988, 1994, 1999, 2005–2006, and 2010–2011 ZDHS surveys that provided updated estimates of basic demographic and health indicators. The primary objective of the 2015 ZDHS survey was to provide current estimates of basic demographic and health indicators. The ZDHS collected information on fertility levels, marriage, sexual activity, fertility preferences, awareness and use of family planning methods, breastfeeding practices, nutritional status of mothers and young children, early childhood mortality, maternal mortality, maternal and child health, knowledge and behaviour related to malaria, HIV/AIDS and other sexually transmitted infections (STIs), smoking, knowledge of cervical cancer, and male circumcision [6].

## Data analysis

Univariate analysis was used to yield descriptive statistics. Principal component analysis (PCA) was used to calculate wealth quintiles, a standardized composite measure combining the cumulative living standard of a household and is based on a household’s ownership of selected assets, such as televisions and bicycles, materials used for housing construction, and types of water access and sanitation facilities. Binary logistic regression approach was used to identify determinants of LLIN ownership and utilization after controlling for other factors. The use of regression models based on ordinary least squares approach is recommended for data that is normally distributed or follows some form of distribution in the dataset [11, 12]. *STATA* version 14 software was used in the analysis for this study.

## Results

Table 1 shows no major changes in demographic characteristics of households sampled between 2010 and 2015 survey cycles. Table 2 shows major socio-economic characteristics investigated in DHS surveys. There are no huge differences in the characteristics of households in 2010 and 2015 surveys. However, owning a bank account and access to clean water indicators improved over the 5-year period (p < 0.001). Table 3 shows that LLIN ownership increased significantly by 42 percentage points from 2010 to 2015. However, Mashonaland East, Harare and Bulawayo experienced decreases in bed net ownership. Table 4 shows a tremendous increase in universal coverage of LLINs between 2010 and 2015. Harare and Matebeleland South surpassed the 100% target for LLIN universal coverage. Matebeleland North and Mashonaland West had lower universal coverages in the country in 2015. Table 5 shows that overall utilization levels of LLINs among children under-five decreased by 11 percentage points between 2010 and 2015. All provinces had decreases in LLIN use among the under-fives except in Harare. Table 6 shows that LLIN usage amongst households followed the same trend with that of the under-fives. Manicaland is the only province where usage increased by 5 percentage points, while Masvingo LLIN usage did not change between 2010 and 2015.

**Table 1:** Demographic characteristics of household members in DHS surveys of 2010 and 2015

Characteristics	2010	2015
Number of participants		
Male	n = 27,225 (64%)	n = 26,987 (62%)
Female	n = 15,423 (36%)	n = 16,719 (38%)
Age in years (mean)		
Male	46	45
Female	47	46
Sex of household head		
Male	n = 20,389 (48%)	n = 20,630 (47%)
Female	n = 22,308 (52%)	n = 23,076 (53%)
Age in years of household head (mean)		
Male	46	45
Female	47	46
Place of residence		
Urban	12,785 (30%)	16,301 (37%)
Rural	29,913 (70%)	27,405 (63%)
Region/province		
Bulawayo	n = 3269	n = 3498
Harare	n = 5712	n = 4536
Manicaland	n = 5128	n = 4905
Mashonaland Central	n = 3841	n = 4723
Mashonaland East	n = 3684	n = 4146
Mashonaland West	n = 4172	n = 4677
Masvingo	n = 4626	n = 4531
Matebeleland North	n = 3646	n = 4071
Matebeleland South	n = 3333	n = 3877
Midlands	n = 5271	n = 4742
Children under 5 years		
Bulawayo	n = 2239	n = 2886
Harare	n = 4581	n = 4201
Manicaland	n = 5787	n = 5678
Mashonaland Central	n = 4 207	n = 4813
Mashonaland East	n = 3234	n = 3672
Mashonaland West	n = 4464	n = 5050
Masvingo	n = 5640	n = 4771
Matebeleland North	n = 4786	n = 4541
Matebeleland South	n = 4065	n = 4008
Midlands	n = 6043	n = 5167
Pregnant women		
Bulawayo	n = 22	n = 43
Harare	n = 72	n = 79
Manicaland	n = 72	n = 78
Mashonaland Central	n = 67	n = 92
Mashonaland East	n = 51	n = 58
Mashonaland West	n = 55	n = 70
Masvingo	n = 75	n = 62
Matebeleland North	n = 38	n = 46
Matebeleland South	n = 37	n = 57
Midlands	n = 88	n = 62

**Table 2:** Socioeconomic characteristics of households based on DHS 2010 and 2015 surveys

Characteristics	2010	2015
Education		
No education	n = 10,894 (26%)	n = 10,293 (24%)
Primary	n = 17,510 (41%)	n = 15,831 (36%)
Secondary	n = 13,049 (31%)	n = 15,136 (35%)
Higher	n = 975 (2%)	n = 2234 (5%)
Mother’s education (children under-five)		
No education	n = 220 (4%)	n = 265 (4%)
Primary	n = 1928 (38%)	n = 2121 (33%)
Secondary	n = 2767 (55%)	n = 3453 (55%)
Higher	n = 118 (2%)	n = 482 (8%)
Pregnant women’s education		
No education	n = 10 (2%)	n = 7 (1%)
Primary	n = 218 (38%)	n = 186 (29%)
Secondary	n = 336 (58%)	n = 414 (64%)
Higher	n = 12 (2%)	n = 39 (6%)
Wealth index		
Poorest	n = 9152 (22%)	n = 7818 (18%)
Poorer	n = 8696 (20%)	n = 7745 (18%)
Middle	n = 8456 (20%)	n = 7800 (18%)
Richer	n = 8184 (19%)	n = 9788 (22%)
Richest	n = 8210 (19%)	n = 10,555 (24%)
Owning a bank		
Yes	n = 27,687 (66%)	n = 31,770 (73%)
No	n = 14,279 (34%)	n = 11,936 (27%)
Access to clean water		
Yes	n = 32,135 (75%)	n = 32,952 (85%)
No	n = 10,560 (25%)	n = 5731 (15%)
Access to sanitation		
Yes	n = 42,491 (99.6%)	n = 43,544 (99.9%)
No	n = 144 (0.4%)	n = 41 (0.1%)
Cooking fuel		
Electricity	n = 12,195 (29%)	n = 12,058 (28%)
Wood	n = 30,042 (70%)	n = 29,017 (66%)
Others	n = 444 (1%)	n = 2631 (6%)

**Table 3:** LLIN ownership patterns amongst households in Zimbabwe in 2010 and 2015

Province	Proportion of households owning at least one LLIN (2010)	Proportion of households owning at least one LLIN (2015)
Manicaland	1034 (15%)	1418 (14%)
Mashonaland Central	912 (13%)	1831 (18%)
Mashonaland East	309 (40%)	1114 (11%)
Mashonaland West	800 (12%)	954 (9%)
Matebeleland North	859 (12%)	1969 (19%)
Matebeleland South	543 (8%)	716 (7%)
Midlands	1082 (16%)	1084 (10%)
Masvingo	642 (9%)	970 (9%)
Harare	441 (6%)	197 (2%)
Bulawayo	350 (5%)	115 (1%)
Total	8945 (21%)	27,702 (63%)

**Table 4:** LLIN utilization patterns amongst children under-5 years in 2010 and 2015

Province	Proportion reporting sleeping under LLIN the night prior 2010 survey	Proportion reporting sleeping under LLIN the night prior 2015 survey
Manicaland	40	37
Mashonaland Central	30	14
Mashonaland East	24	25
Mashonaland West	19	18
Matebeleland North	42	22
Matebeleland South	43	18
Midlands	39	29
Masvingo	32	18
Harare	8	19
Bulawayo	31	16
Average	31%	22%

**Table 5:** LLIN utilization patterns amongst households in 2010 and 2015

Province	Proportion reporting sleeping under LLIN the night prior 2010 survey	Proportion reporting sleeping under LLIN the night prior 2015 survey
Manicaland	25	30
Mashonaland Central	31	11
Mashonaland East	22	20
Mashonaland West	18	15
Matebeleland North	37	18
Matebeleland South	38	16
Midlands	32	15
Masvingo	22	22
Harare	17	16
Bulawayo	23	15
Average	27%	18%

**Table 6:** Factors associated with LLIN ownership amongst households in Zimbabwe in 2015

Variable	OR [95% CI] 2015	p-value
Number of household members	1.000 (0.981–1.020)	0.956
Region with low malaria incidence	0.921 (0.906–0.934)	*<* *0.001*
Rural residence	1.28 (1.091–1.498)	*0.002*
Electricity	0.804 (0.682–0.948)	*0.009*
Radio	1.119 (1.026–1.219)	*<* *0.001*
TV	1.081 (0.950–1.23)	0.238
Floor	0.999 (0.9931–1.006)	0.915
Wall	0.994 (0.986–1.000)	0.080
Finished roof	0.991 (0.984–0.998)	*0.021*
Number of rooms for sleeping	0.966 (0.922–1.012)	0.148
Male head of household	0.865 (0.794–0.942)	*0.001*
Head of household> 30 years	1.005 (1.002–1.009)	*0.001*
Telephone	0.763 (0.629–0.924)	*0.006*
Electricity as cooking energy	0.977 (0.957–0.997)	*0.024*
Mobile	1.39 (1.19–1.62)	*<* *0.001*
Owning bank account	1.15 (1.02–1.29)	*0.017*
Sprayed against mosquitoes	1.126 (1.067–1.187)	*<* *0.001*
Rich or richer	1.19 (1.10–1.29))	*<* *0.001*
Decorder	1.22 (1.066–1.39)	*<* *0.004*
Education level	0.922 (0.861–0.988)	0.063
Pregnancy	0.853 (0.929–2.42)	0.097

Italics shows statistical significance

In 2015 region/province, type of place of residence, availability of electricity, radio, roof type, gender of head of household, having telephone, type of cooking fuel, presence of mobile phone, owning a bank account, IRS spraying in the previous 12 months, wealth index, and satellite television decorder were independently associated with net ownership amongst households.

Table 7 shows that type of place of residence, age of household head, type of cooking fuel, IRS in previous 12 months, and pregnancy were associated with LLIN utilization.

**Table 7:** Factors associated with LLIN utilization amongst households in Zimbabwe in 2015

Variable	OR [95% CI] 2015	p-value
Number of household members	0.966 (0.928–1.004)	0.080
Region	0.977 (0.949–1.006)	0.115
Urban residence	0.571 (0.426–0.764)	*<* *0.001*
Electricity	1.1639 (0.851–1.587)	0.343
Radio	0.983 (0.841–1.114)	0.825
TV	1.033 (0.820–1.301)	0.782
Floor	0.992 (0.979–1.004)	0.219
Wall	1.005 (0.992–1.019)	0.459
Roof	0.993 (0.79–1.006)	0.290
Number of rooms for sleeping	1.052 (0.964–1.14)	0.253
Sex of head of household	0.968 (0.825–1.13)	0.687
Head of household < 30 years	0.986 (0.979–0.993)	*<* *0.001*
Telephone	1.055 (0.710–1.568)	0.790
Electricity as cooking energy	0.957 (0.917–1.000)	*0.048*
Mobile	1.097 (0.809–1.488)	0.551
Owning bank account	0.881 (0.714–1.089)	0.243
Sprayed against mosquitoes	1.160 (1.074–1.252)	*<* *0.001*
Rich or richer	0.946 (0.811–1.10)	0.479
Decorder	0.879 (0.693–1.117)	0.292
Education level	0.872 (0.757–1.005)	0.059
Pregnancy	0.623 (0.432–0.898)	*0.011*

Italics shows statistical significance

## Discussion

This study showed no major changes in demographic characteristics of households sampled between 2010 and 2015 survey cycles. LLIN ownership increased significantly by 42 percentage points from 2010 to 2015. There was a tremendous increase in universal coverage of LLINs between 2010 and 2015. The overall utilization levels of LLINs among children under-five decreased by 11 percentage points between 2010 and 2015. LLIN usage among households followed the same trend with that of the under-fives.

Despite significant strides to improve coverage and access of LLINs using mass campaigns and routine distributions, LLIN utilization has been decreasing in transmission regions of Zimbabwe. Utilization amongst children under-five decreased from 32% in 2010 to 21% in 2015. In the general population, utilization also decreased from 27% in 2010 to 18% in 2015. It is not clear what could be driving the low utilization patterns, however, perceptions of risk, human behaviour, lack of knowledge, and inadequate SBCC may be contributing to the trends [13, 14]. Some studies have indicated that education levels, material resources, good quality housing, access to nets, and community outreaches improve access and utilization of LLINs [15, 16]. This study also noted some similar findings to the factors that are associated with LLIN ownership and utilization. The factors identified in the DHS survey are predominantly of socio-economic nature and this requires intersectoral approaches in order to address the barriers to utilizing LLIN to prevent malaria. Tassew et al. [17] in their study noted that plastered walls or cement walls were associated with LLIN utilization, however, in this present study ownership was associated only with walls.

This study revealed that the floor, walls and roof were independent predictors of LLIN ownership but not utilization and this finding is supported by other studies [17, 18]. In these recent studies, researchers’ findings were related to factors associated with net utilization. However, in this study the identified factors were associated with net ownership and not utilization. Kitidamrongsuk et al. [13] noted that previous malaria infection and irregular use of electric fans were associated with LLIN utilization; however, this present study noted that having electricity in a household was associated with LLIN ownership but not utilization in 2015. Xu et al. [15] in their study noted that household income, house type and head of household knowledge about bed nets preventing malaria were predictors of usage, however, this present analysis has shown that housing type (walls, floors, roof), wealth index, religion and head of household’s gender were associated with LLIN ownership but not utilization. Factors associated with LLIN utilization identified were age of head of household, type of place of residence, type of cooking fuel, history of spraying, and pregnancy. In the Zimbabwean context there were more factors identified as being associated with LLIN ownership than utilization and these relationships may need to be understood more deeply as a first step to addressing the declining usage of LLINs. It is also plausible that the declining utilization patterns may be associated with underlying socio-economic issues.

In 2015 survey, the association of history of spraying and pregnancy with LLIN utilization were supported by the findings of Watanabe et al. [16] findings, especially on perceived risk and malaria-prevention benefit. In Zimbabwe, as malaria incidence plummets and some districts are transitioning to pre-/elimination it is possible that perceived risk and malaria-prevention benefit may be getting lower hence driving low LLIN utilization. This study did not identify significance of education levels and household income on both ownership and utilization of nets as reported by Idowu et al. [19] in Nigeria. Usage of LLIN amongst children under-five was higher than the general population and this finding is not supported by Wanzira et al. [20], who reported that children between 6 and 14 years were more likely to use an LLIN than children under-five in Uganda. Some of the findings in this study support the results of Moon et al. [21] who reported that larger households, larger monthly income and having electricity were associated with LLIN utilization. The outcomes of this analysis revealed that having electricity was associated with ownership and not utilization in 2015.

With global and regional blocks having adopted a malaria elimination agenda it becomes imperative to understand factors that relate to vector control interventions. The successes or failures of any intervention are hinged on understanding the factors that are associated with its uptake or utilization. Most heath interventions being prescriptive and driven by donor agencies tend to ignore certain important aspects about the interests of the targeted beneficiaries and some programmes have failed because of some of these issues [22–24]. While several studies have shown determinants of utilization [13–21], the present study revealed some factors associated with ownership, which need to be understood independently. Some of the factors identified in this study need to be constantly monitored at different levels of the continuum of interventions. Over time interventions need to be reviewed and adjusted based on evidence due to the dynamics of human life and public health issues.

This study had its share of limitations; firstly the analysis was based on data from cross sectional surveys which cannot be used to infer causal relationships. Furthermore, LLIN ownership and utilization were based on self-reporting, which is subject to bias. However, this study had its strengths; mainly that it was based on data from nationwide surveys and very big sample sizes had been used which resulted in precise estimates.

## Conclusions

This study revealed increasing LLIN coverage but decreasing usage in malaria transmission regions of Zimbabwe. The findings of this study are a good starting point for further research and exploratory work in Zimbabwe and other similar contexts. Strengthening of LLIN campaigns, SBCC interventions and programme routine monitoring are recommended to improve LLIN usage in malaria-transmission regions.

## Data Availability

The datasets used and/or analysed may be availed by ICF International on justifiable request.

## Abbreviations

IRS: indoor residual spraying
LLIN: long-lasting insecticidal nets
MIS: Malaria Indicator Survey
MoHCC: Ministry of Health and Child Care
MPR: malaria programme review
NGO: non-governmental organization
SBCC: social and behaviour change communication
USAID: United States of America International Development
